# The increase in cobalt release in metal-on-polyethylene hip bearings in tests with third body abrasives

**DOI:** 10.1177/0954411915595433

**Published:** 2015-09

**Authors:** Danielle de Villiers, Alison Traynor, Simon N Collins, Julia C Shelton

**Affiliations:** 1 School of Engineering and Materials Science, Queen Mary University of London, London, UK; 2 Corin Ltd, Cirencester, UK

**Keywords:** Wear testing, hip replacements, cobalt, polyethylene

## Abstract

Hypersensitivity reactions in patients receiving metal-on-metal hip replacements have been attributed to corrosion products as observed by elevated cobalt and chromium ions in the blood. Although the majority of cases are reported in metal-on-metal, incidences of these reactions have been reported in the metal-on-polyethylene patient population. To date, no in vitro study has considered cobalt release for this bearing combination. This study considered four 28 mm and seven 52 mm diameter metal-on-polyethylene bearings tested following ISO standard hip simulator conditions as well as under established abrasive conditions. These tests showed measurable cobalt in all bearings under standard conditions. Cobalt release, as well as polyethylene wear, increased with diameter, increasing from 52 to 255 ppb. The introduction of bone cement particles into the articulation doubled polyethylene wear and cobalt release while alumina particles produced significant damage on the heads demonstrated by cobalt levels of 70,700 ppb and an increased polyethylene wear from a mean value of 9–160 mm^3^/mc. Cobalt release was indicative of head damage and correlated with polyethylene wear at the next gravimetric interval. The removal of third body particles resulted in continued elevated cobalt levels in the 52 mm diameter bearings tested with alumina compared to standard conditions but the bearings tested with bone cement particles returned to standard levels. The polyethylene wear in the bone cement tested bearings also recovered to standard levels, although the alumina tested bearings continued to wear at a higher rate of 475 mm^3^/mc. Cobalt release was shown to occur in metal-on-polyethylene bearings indicating damage to the metal head resulting in increased polyethylene wear. While large diameter metal-on-polyethylene bearings may provide an increased range of motion and a reduced dislocation risk, increased levels of cobalt are likely to be released and this needs to be fully considered before being widely adopted.

## Introduction

Improvements in polyethylene quality have focused on the introduction of crosslinking to reduce wear and post processing methods such as remelting and annealing or, more recently, anti-oxidants incorporated into the polyethylene to reduce oxidation.^1^ These developments in polyethylene have led to the proposal for larger diameters in total hip replacement components, historically limited in this bearing combination due to the observed relationship between polyethylene wear and diameter.^2^ However, larger diameters (greater than 44 mm) have been reported to lead to an increased rate of revision^3^ and in vitro studies have suggested that an increased head size increases the torque between the head–taper interface possibly increasing corrosion.^4^ A larger diameter head on a taper also introduces a larger lever arm which can create toggling leading to failure of the implant.^5^ Metal wear and corrosion products are believed to be responsible for hypersensitivity and pseudotumours as well as other types of adverse local tissue reactions frequently reported in patients receiving metal-on-metal hip replacements.^6–8^ Recently, there have been reports of pseudotumours in the metal-on-polyethylene patient population^9,10^ suggesting metal wear and corrosion or a combination of these, either from the bearing surface or at the head–neck taper interface, may occur.

The bearing surfaces of metal-on-polyethylene retrievals have widely been shown to present with scratching and embedding of particles in the metal and polyethylene surfaces.^11–14^ The addition of third body particles has been an established adverse hip simulator wear model^15,16^ but has primarily focused on the influence of these particles on polyethylene wear. Metal-on-metal tests, however, have shown that metal bearings are susceptible to abrasive wear^17^ and the observation of scratches on the metal head following retrieval suggests that material may be removed from the bearing surfaces. Similarly, increases in cobalt concentration in the blood of patients with metal-on-polyethylene bearings compared to controls (who have not received a total hip replacement) and the consequent formation of pseudotumours have also been reported, although the current opinion is that this likely originates from the head–taper interface.^18^

The aim of this study was to investigate the potential for cobalt release in clinically relevant 28 mm diameter metal-on-polyethylene bearings under standard and adverse third body hip simulator conditions as well as an exploration of larger, prototype 52 mm diameter bearings.

## Materials and methods

Four 28 mm internal diameter and seven 52 mm internal diameter (external diameters of 57.75 and 58.25 mm, respectively) 0.1 wt% vitamin-E blended polyethylene liners highly cross-linked at 120 kGy and mechanically annealed (Corin Ltd, UK) were paired with CoCrMo heads (cast solution annealed and hot isostatically pressed) with clearances of 100–400 µm in the 28 mm diameter and 300–700 µm in the 52 mm diameter components. All bearings were tested on an eight station orbital hip simulator in an anatomical position (MTS Systems, USA) for 1 million cycles following loading according to ISO 14242-3:2009^19^ using 25% newborn calf serum (Batch number 11E191; Sigma-Aldrich, USA) diluted with pure deionised water to give a protein concentration of 17 g/L as a lubricant. Three 52 mm bearings were tested further to 5 million cycles under this condition. Following standard test conditions, 1 million cycles of third body testing was conducted with either bone cement (in bearings tested to 1 million cycles under standard conditions) or alumina particles (bearings tested to 5 million cycles) (Table 1). These particles were chosen as third body abrasives as they have both been used in previous studies^15,16,20–22^ and reported to increase polyethylene wear and produce damage in the metal heads comparable to those reported at retrieval. The bone cement particles were from pre-polymerised powder (Simplex P, Stryker, USA) comprising particles sized between 0.3 and 138.0 µm. This showed a bimodal distribution with larger particles (mode: 24.6 µm) of methyl methacrylate styrene and smaller particles (mode: 1.8 µm) of polymethylmethacrylate (PMMA) and barium sulphate. The powder was added directly to the test fluid at a concentration of 5 mg/mL, the minimum concentration reported previously^15^ to produce an increase in wear rate. The alumina particles were smaller, ranging in size from 0.7 to 5.3 µm with bimodal distribution modes of 0.8 and 3.1 µm. These particles were added at a concentration of 0.15 mg/mL as described by Bragdon et al.^16^ in order to achieve clinically relevant damage in the form of Ra and Rz observed at retrieval, although the damage mechanism itself was not clinically relevant. The testing of 52 mm diameter bearings with alumina third body particles was intended to represent an extreme condition. A final 1 million cycles of testing was conducted with these third body particles removed from the test fluid to determine the influence of any damage initiated by the third body particles.

**Table 1.** Summary of third body test conditions.

Bearing internal diameter (mm)	Number of bearings	Third body used	Concentration of third body (mg/mL)
28	4	Bone cement	5
52	4	Bone cement	5
52	3	Alumina	0.15

Wear was determined gravimetrically for the polyethylene liners and converted to volumetric loss assuming a density of 0.942 g/cm^3^. Unloaded soak controls of the liners were subjected to similar environmental factors as the bearings tested and their mass recorded in order to correct for water absorption. Cobalt release into the test fluid was determined using graphite furnace atomic absorption spectrometry (GFAAS) (A SpectrAA, 220FFS atomic absorption spectrometer with GTA-110 autosampler; Varian, UK) as described elsewhere.^23^ Surface roughness of the heads before and after each test phase was determined using an optical surface profilometer (Bruker, USA) using a green narrow band filter and a 20× magnification lens. Five measurements of each head were taken: one at the apex of the head and four measurements around the expected worn area of the head. Statistical significance was determined using a Student’s t-test (p < 0.05) for all tests.

## Results

**Figure 1.**
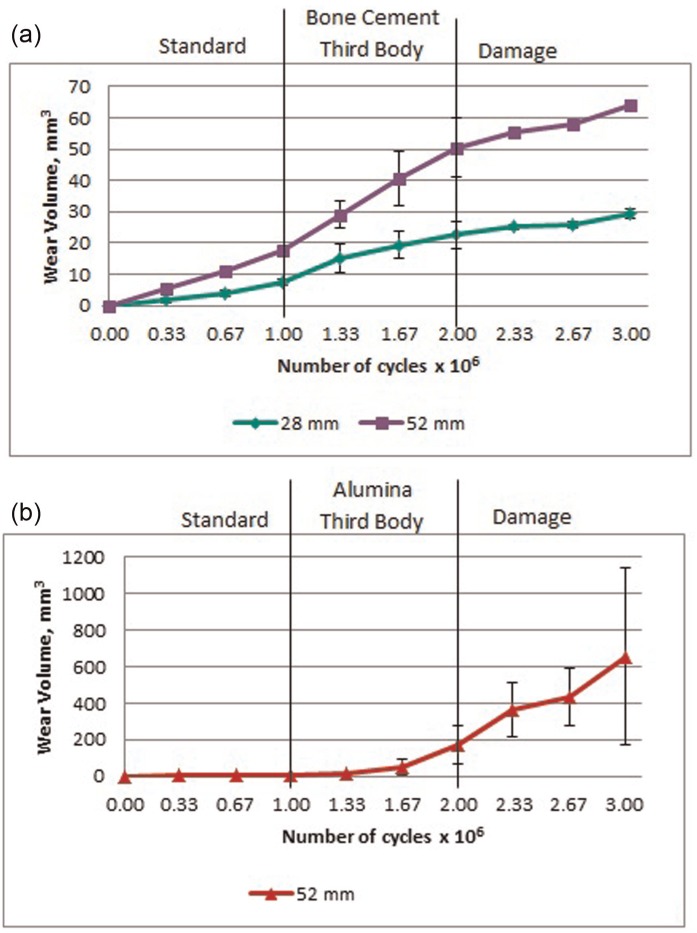
Mean cumulative wear volume (± 1 standard deviation) of (a) 28 and 52 mm diameter metal-on-polyethylene bearings tested with bone cement as a third body and (b) 52 mm diameter bearings tested with alumina as a third body.

**Figure 2.**
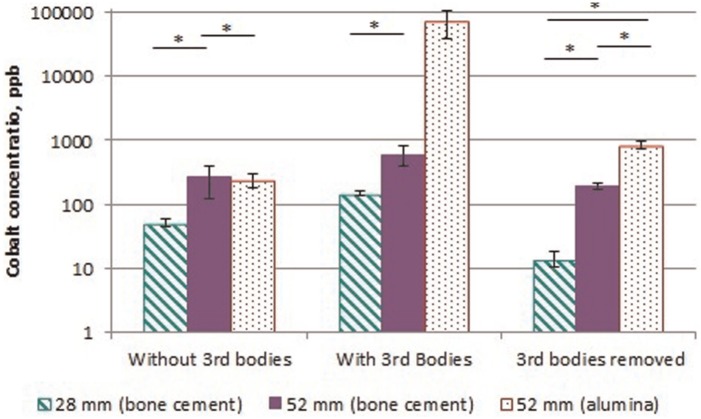
Mean cumulative cobalt levels (±1 standard deviation) in bearings after 1 million cycles under each test condition (the symbol ‘*’ indicates significant difference p < 0.05).

Under standard wear conditions, no higher wear rate run-in was observed over 5 million cycles of testing as would have occurred in standard polyethylene. The polyethylene wear rate of the 28 mm diameter bearings (7.4 ± 1.1 mm^3^/mc) was approximately half that of the 52 mm diameters (14.3 ± 4.4 mm^3^/mc), Figure 1. The influence of diameter was also noticeable in cobalt release with the 52 mm diameter bearings producing 255 ± 104 ppb over 1 million cycles, significantly greater than 52 ± 7 ppb from the 28 mm diameter (Figure 2). There was no change in the surface roughness parameters of any of the heads between 0 and 1 million cycles.

**Figure 3.**
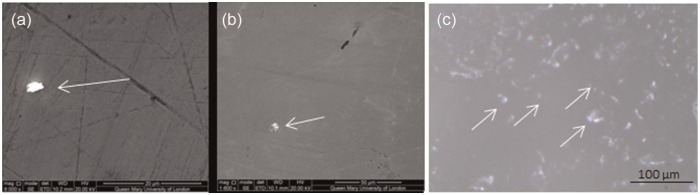
Bone cement particles (arrows) embedded in (a) 28 mm diameter head, (b) 52 mm diameter head and (c) 52 mm diameter liner.

The addition of bone cement to the test fluid significantly increased polyethylene wear, approximately doubling the rate in both diameters to 14.9 ± 4.0 and 32.8 ± 9.9 mm^3^/mc in the 28 and 52 mm diameter bearings, respectively, over 1 mc. However, the wear response of the diameters to the introduction of bone cement was different during this period. The 28 mm diameter bearings produced an initial high rate of wear over the first third of a million cycles but this reduced with the continuation of testing. The 52 mm diameter bearings, however, showed no recovery, continuing to consistently wear at double the standard conditions rate throughout the test duration. The abrasives did not change the surface roughness of the heads yet the cobalt released into the test fluid doubled. Embedding of the bone cement particles was observed in both the polyethylene and metal bearing surfaces following their introduction and 1 million cycles of testing (Figure 3).

**Figure 4.**
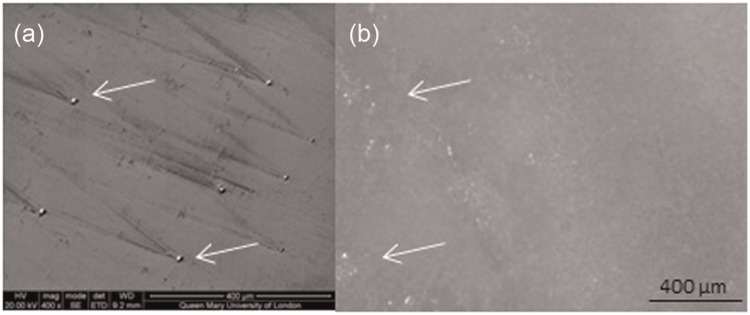
Alumina particles (arrows) embedded in (a) 52 mm diameter head and (b) 52 mm diameter liner.

**Figure 5.**
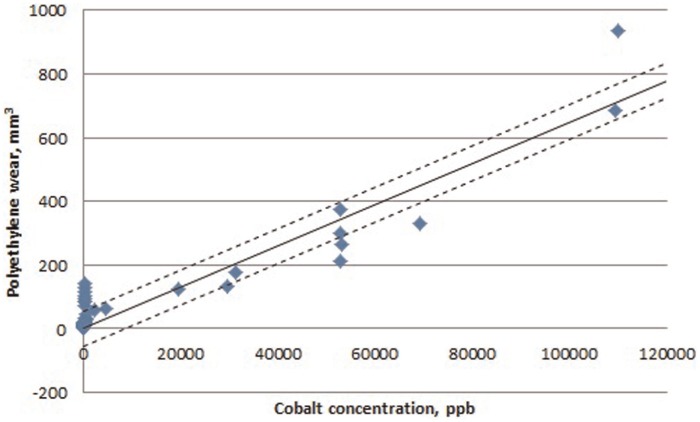
Linear relationship between cobalt release at a gravimetric interval and polyethylene wear at the next interval in all bearings under all test conditions indicating an ability to predict polyethylene wear based on cobalt release.

The three 52 mm diameter bearings which were tested with alumina particles as abrasives exhibited an 11-fold increase in polyethylene wear to 159.8 ± 108.6 mm^3^/mc. The wear rate increased with each gravimetric wear interval with a wear rate of 368.7 ± 202.3 mm^3^/mc between 0.67 and 1.00 mc. The cobalt levels also significantly increased producing 70,700 ± 32,500 ppb over the 1 million cycles of testing, an increase of 270 times compared to standard conditions. The elevated cobalt levels in the test fluid indicated removal of the metal from the head bearing surface which was confirmed through the observation of an increased Rz roughness to 2.73 ± 1.13 µm (Table 2). Embedding of the third body particles was also observed in both the metal and polyethylene surfaces (Figure 4). In the bearings tested with alumina particles, a relationship between cobalt levels and polyethylene wear was established; the cobalt released at a gravimetric interval would indicate the polyethylene wear at the next gravimetric interval with a Pearson’s correlation of 0.99. Throughout all testing in all bearings, a relationship between cobalt release and subsequent polyethylene wear was established but with a lower correlation of 0.94 (Figure 5). This regression had a standard error of ±55 mm^3^ of polyethylene wear and therefore was more appropriate in predicting large volumes of polyethylene wear such as that which occurred during adverse third body conditions.

**Table 2.** Mean surface roughness (±1 SD) measurements of 28 and 52 mm diameter heads throughout testing.

Rz (µm)	n	0 mc	1 mc	After third body	After damage
28 mm (bone cement)	4	2.66 ± 0.69	1.86 ± 0.78	1.80 ± 0.73	1.74 ± 0.81
52 mm (bone cement)	4	1.65 ± 0.72	1.59 ± 0.70	1.82 ± 0.65	1.83 ± 0.66
52 mm (alumina)	3	1.65 ± 0.72	1.59 ± 0.70	2.73 ± 1.13	3.01 ± 1.61

SD: standard deviation.

Further testing, with the removal of third body particles from the test fluid, revealed the differences in the damage generated by bone cement and alumina particles. The bearings tested with bone cement appeared to recover and wear rates and cobalt release both reduced to standard condition levels, although a significant decrease in polyethylene wear was observed in the 28 mm diameter bearings. The damage and embedded particles in the bearings tested with alumina particles proved to be more extensive, resulting in higher polyethylene wear, 474.9 ± 470.4 mm^3^/mc, than during third body testing. Large variation was observed between the wear rates of the three bearings, possibly as different degrees of damage were produced. The cobalt release was also higher than that produced under standard conditions but reduced from third body testing and no further damage on the heads in the form of surface roughness changes was observed.

## Discussion

This study has shown that vitamin-E blended highly cross-linked polyethylene at 28 and 52 mm diameters can produce wear rates comparable to 5 ± 2 and 10 ± 2 mm^3^/mc reported in 28 and 36 mm cross-linked polyethylene bearings, respectively,^24,25^ suggesting that larger diameter polyethylene liners could be used clinically without the risk of excessive wear. The use of a liner with a larger inner diameter necessarily requires a thinner wall, which in turn can increase the stress within the polyethylene, possibly increasing the rate of fracture.^26^ Fracture of the liners was not observed in this study and other in vitro studies have suggested that liner thickness does not influence wear.^27^ The increase in cobalt release when utilising a larger metal head may be, however, a limiting factor when considering size.

This study has shown that cobalt release from a CoCrMo femoral head occurs, but the origin of this cobalt remains unclear as the heads were mounted on stainless steel tapers which were not isolated. Cobalt release was observed to increase with increasing diameter, which may be due to higher torque and/or an increased lever arm experienced on the taper junction with the larger diameter^4,5^ or may be due to the increased surface area of the larger heads. In a well behaving metal-on-polyethylene bearing, such as in standard hip simulator conditions representing correct positioning, well-connected tapers and a normal walking gait, the cobalt release in the 52 mm diameter bearings (255 ppb) was significantly lower than the 8904 ppb reported in metal-on-metal studies.^28^ These values are larger than that reported clinically (1–5 ppb) in the blood as the cobalt measured resulted from an accumulation of cobalt released over the test period. Currently, it is unknown at what threshold cobalt levels (released in ionic and particulate form) produce hypersensitivity reactions and; therefore, it is unclear as to whether the body would be able to safely process the cobalt released in large diameter metal-on-polyethylene bearings.

The introduction of clinically relevant abrasive particles (bone cement) to the test fluid of metal-on-polyethylene bearings doubled the polyethylene wear rate as well as the cobalt levels. No change was observed in the surface roughness of the heads under this test condition which suggests that the bone cement may have scratched the metal head surfaces but not enough to prevent repolishing. The wear response to the addition of bone cement third body particles to the test fluid highlighted differences in diameter. Although initially more severe in 28 mm diameter bearings with a threefold increase in the first third of a million cycles, the wear following this reduced to 14.9 mm^3^/mc. The 52 mm diameter bearings showed no recovery possibly as a result of their higher clearance in these bearings. The higher clearance may have allowed the introduction of greater numbers of third body particles between the articulating surfaces. Sorimachi et al.^21^ reported greater adherence of PMMA to larger diameter polyethylene liners compared to smaller liners, and so this may be a diameter or surface area effect. The wear rates reported in this work for both diameter bearings tested with bone cement were lower than previous studies using cross-linked polyethylene at the same concentration,^15,29^ indicating that this generation of polyethylene is more resistant to abrasion than previous forms.

The wear rate in the bearings tested with alumina particles increased 11-fold compared to standard wear conditions and was shown to further increase with testing duration suggesting that the conditions of testing became more severe possibly due to increasing surface damage on the bearing surface with each gravimetric interval. The wear rate reported in this study was comparable to that observed in 28 mm diameter conventional polyethylene bearings tested under the same conditions.^22^ The wear rate recorded in this work is higher than that reported for cross-linked polyethylene^16,22^ but the diameters used in these studies were small at 28 and 36 mm. The alumina particles generated significant damage in the heads, observed through changes in both the surface roughness of the heads and the cobalt released into the test fluid. The cobalt levels measured during 1 million cycles under this test condition (70,700 ppb) were greater than that reported in metal-on-metal bearings tested using the same simulator.^28^ The cobalt release increased with each gravimetric measurement interval, suggesting increasing damage which also led to the increasing polyethylene wear observed. The high cobalt levels generated through the alumina abrasive condition is unlikely to be encountered clinically, although instances of ceramic head fracture in vivo have been reported leading to substantial damage of the polyethylene liner.^30–33^ The damage generated in the heads produced a comparable surface roughness to that observed and commonly reported in retrieved components.^12^

After third body testing, embedding of bone cement and alumina was observed in the heads and liners despite rigorous cleaning which has been acknowledged to remove some of the embedded particles and therefore reduce the surface roughness.^20^ The embedding of third body particles has been reported in metal and polyethylene surfaces in a pin-on-plate study with embedding dependent on both the hardness of the abrasive particles and the bearing surface.^34^ As bone cement is softer than alumina, less deep embedding may have occurred, although this was not quantified. The continuation of testing following the removal of the third body particles from the test fluid showed the bearings tested with bone cement to return to standard conditions indicating that the damage produced was either transient or insufficient to generate wear. Previous studies have reported a reduction in polyethylene wear following bone cement testing, suggesting that the embedded bone cement may act as hard islands in the polyethylene.^15,20^ Adherence of bone cement to metal bearing surfaces has been proposed for the prevention of cobalt release^35^ and could explain the reduction in cobalt measured during the final million cycles of testing.

The damage generated by alumina particles embedded into the heads and/or liners led to further increase in polyethylene wear higher than that reported in the third body condition. Two of the three bearings appeared to show some recovery, but the highest wearing and, therefore, most damaged bearing continued to wear highly suggesting there is a level of damage below which is possible for the bearings to recover. The high wear rate observed is greater than reports from controlled scratched heads.^36–38^ The surface roughness of the heads reported in this study was comparable or lower than the literature,^37–39^ suggesting that the embedding of particles into the head may have been the main factor leading to an increase in wear. The cobalt released during this testing was approximately four times greater than that reported under standard conditions, possibly due to the embedded alumina in the polyethylene continuing to scratch the heads or an increased surface area due to the increased roughness.

Several clinical reports have shown raised blood ions in patients with metal-on-polyethylene bearings in comparison with patients without implants,^40–42^ but this study is the first to measure cobalt release in vitro for this bearing combination. The correlation between metallic wear and cobalt release in metal-on-metal simulator tests has been reported^28,43^ and this study proposes a relationship that occurs in metal-on-polyethylene. As polyethylene wear is dependent on the condition of the head^37^ and cobalt release is indicative of metal wear, monitoring of the cobalt levels in the urine or blood of these patients may also be important. This study suggests that the origin of at least some of the cobalt measured in this study was released from the bearing surface and it was not wholly derived from the taper interface. In patients with a metal-on-polyethylene implant, cobalt levels may therefore allow for the detection of hypersensitivity reactions as well as provide an early detection for likely increasing polyethylene wear.
